# The 2025 ISCB Innovator Award—Dr Fabian Theis

**DOI:** 10.1093/bioinformatics/btaf260

**Published:** 2025-07-15

**Authors:** Mallory L Wiper

**Affiliations:** The International Society for Computational Biology, Leesburg, Virginia, United States

**Figure:**
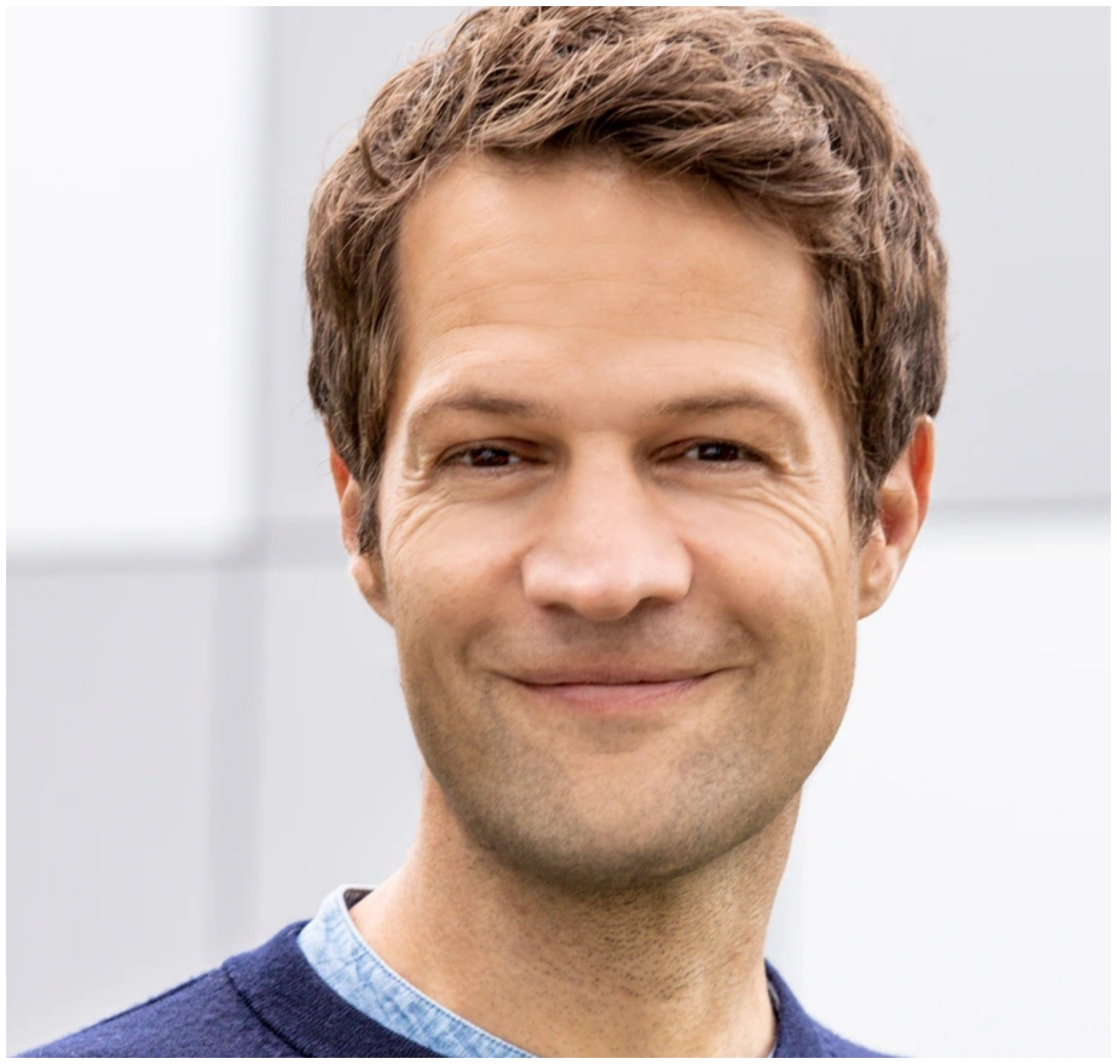


The ISCB Innovator Award is presented annually to a leading scientist who not only makes progressive contributions to computational biology but who also consistently pursues unexplored directions in the field. At this year’s, 33rd Annual Intelligent Systems for Molecular Biology conference and the 24th European Conference on Computational Biology, the International Society for Computational Biology is honored to present the Innovator Award to Dr Fabian Theis.

## From LEGO to computational biology

Theis tracks his interest in science back to childhood, stemming from his love of building elaborate LEGO constructions—a passion that took a transformative turn around age 9 when he received an early personal computer. Unlike modern PCs, however, the PC Theis received did not have any games, but he saw that as a problem to solve and learned programming to create his own.

This challenge of constructing games on his PC sparked a fascination with the logic, rigidity, structure, and algorithmic thinking involved in computer science. The fascination with logic and structure only deepened his passion for computer science, but it did little to spark an interest in biology. Theis recalls hating biology in school because of how messy and unpredictable it was. Biology did not have the clear, logical flow and firm laws of subjects like math and physics.

It was not until the late stages of his PhD that Theis came around to biology. Theis realized that when computational methods and approaches were utilized, they could bring structure and order to occasionally chaotic biological data. This shift in understanding ultimately led Theis to the field of computational biology where he applied machine learning to analyze biological complexity to reveal patterns and insights that might be missed by traditional experiments.

## Guided by great mentors

Theis credits several key mentors with shaping his academic and professional journey. Chief among them is his PhD supervisor Elmar Lang under whose guidance Theis shifted from a PhD in mathematics to one in biophysics. Through Lang, Theis transitioned from theoretical to applied research, gaining exposure to statistical learning, signal processing, and machine learning applications, and discovering the value of scientific publications along the way.

Following his PhD, Theis set up a junior lab in Munich after being hired by Hans-Werner Mewes who had set up the first bioinformatics course in Munich. Mewes was instrumental in helping bridge the gap between computation and biology for Theis, helping him get into the field on a larger scale and connecting him with collaborators in biology.

Friends and colleagues have also been important to Theis’s growth in computational biology. He noted Aviv Regev and Dana Pe’er as influential voices in the field, recalling that Pe’er’s Innovator Award Keynote address at ISMB/ECCB 2023 blew him away with the way she told the story of her research, weaving it together in a compelling narrative, demonstrating the importance of storytelling in science. Theis has also learned a lot from mentor and friend Matthias Tschöp, the current head of Helmholtz Munich. Under Tschöp’s guidance, Theis gained valuable experience in organizing large scientific teams and played a key role in establishing the Helmholtz Computational Health Center, which he now directs. The center has grown to include more than 40 PIs and over 400 scientists focused on AI-based computational biology and biomedicine.

Last but not least, Theis highlighted his students as some of the greatest influences on his career, bringing fresh perspectives and innovative ideas to the lab. He shared that mentoring them and helping guide their academic journeys is what keeps him motivated each day because their curiosity and creativity continue to push his research in new and exciting directions.

## Growing as a leader and mentor

Theis recalls the transition from post-doc to principal investigator (PI) as being hard for him because he had to let go of direct problem-solving and doing everything himself. As a post-doc, he was used to working independently and solving problems quickly. As a PI, he’s had to be hands-off with problem-solving when it comes to his *student’s* projects, recognizing that it’s more beneficial for them to struggle a little and develop their own approaches without too much interference. Over time, he’s found that having different people working on problems in different ways not only benefits the students but also demonstrates trust in his team and ultimately helps to scale the impact of the lab’s work.

When it comes to being a mentor, Theis credits much of his development to open conversations with peers—especially those who are one step ahead in their own careers—and to learning from the best practices he observed around him. Rather than assuming he had all the answers, Theis valued listening, adapting, and learning from others to shape a mentorship style focused on collaboration, communication, and interdisciplinary training.

An example of this mentoring style is Theis’s “Y model” approach to training PhDs. Each PhD student has one mentor focused on computational and methodological skills, and one mentor focused on biological and experimental applications. This collaborative training ensures that each student has a well-rounded understanding of both theory and practice.

Theis also strongly encourages students to explore topics of interest before settling on a single focus for their research, noting that completing a PhD can be a bit of a scary thing, but he aims to make sure his students have fun during that time, too! He wants them to have the freedom to experiment, take risks, and develop their own research identity.

## Unexpected discoveries and new frontiers

One of the most unexpected successes in Theis’s research career came when Alex Wolf, a post-doc in his lab, proposed improving the previously slow and inefficient single-cell analysis process through automation. Specifically, Wolf suggested they develop a structured framework for handling and analyzing the data, and from this suggestion, ScanPy was created. ScanPy is now a widely used standard for such analysis and has been downloaded over 5 million times!

The experience in developing this software reinforced for Theis the importance of software engineering in scientific research which has since shifted his focus to prioritizing computational tools as a crucial part of biological discovery.

In addition to the creation of ScanPy, he also reflected on the early days of whole-cell modeling in the 2010s, which at the time fell short due to limited data and computational resources. Today, however, advances in AI and data integration are reviving these approaches, making predictive whole-cell models far more feasible.

Currently, Theis is most fascinated by foundation models in biology—these models are like large language models, but for biological data. His team is exploring how these models can be used to make predictions and guide experimental design with the hope that the foundation models will ultimately optimize not only how scientists interpret experimental output but also how they design experiments. This concept of models being continuously refined by new data and creating self-improving research frameworks is an exciting avenue that Theis is keen to explore!

## Reflecting on winning the 2025 ISCB Innovator Award

Theis said he was “honored and very humbled” to be named the 2025 ISCB Innovator Award recipient and to be recognized alongside some of the stars in the field. He also emphasized that this was not an award he earned on his own, but that it reflected the work carried out by his entire team, highlighting the “talented, fantastic students [he has] the honor of working with.”

